# Fat mass and obesity-associated factor (FTO)-mediated N6-methyladenosine regulates spermatogenesis in an age-dependent manner

**DOI:** 10.1016/j.jbc.2023.104783

**Published:** 2023-05-03

**Authors:** Yifei Wu, Jincheng Li, Chenmeijie Li, Shuai Lu, Xiaoyu Wei, Yang Li, Wenjuan Xia, Chunfeng Qian, Zihang Wang, Mingxi Liu, Yayun Gu, Boxian Huang, Yueqiu Tan, Zhibin Hu

**Affiliations:** 1 State Key Laboratory of Reproductive Medicine, Nanjing Medical University, Nanjing, Jiangsu, China; 2 Department of Epidemiology, Center for Global Health, School of Public Health, Nanjing Medical University, Nanjing, Jiangsu, China; 3 State Key Laboratory of Reproductive Medicine, Affiliated Suzhou Hospital of Nanjing Medical University, Suzhou Municipal Hospital, Suzhou, China; 4 Clinical Research Center for Reproduction and Genetics in Hunan Province, Reproductive and Genetic Hospital of CITIC-Xiangya, Changsha, China

**Keywords:** FTO, m6A, spermatogenesis, age-dependent, androgen receptor

## Abstract

N6-methyladenosine (m6A) is the most prevalent reversible RNA modification in the mammalian transcriptome. It has recently been demonstrated that m6A is crucial for male germline development. Fat mass and obesity-associated factor (FTO), a known m6A demethylase, is widely expressed in human and mouse tissues and is involved in manifold biological processes and human diseases. However, the function of FTO in spermatogenesis and male fertility remains poorly understood. Here, we generated an *Fto* knockout mouse model using CRISPR/Cas9-mediated genome editing techniques to address this knowledge gap. Remarkably, we found that loss of *Fto* in mice caused spermatogenesis defects in an age-dependent manner, resulting from the attenuated proliferation ability of undifferentiated spermatogonia and increased male germ cell apoptosis. Further research showed that FTO plays a vital role in the modulation of spermatogenesis and Leydig cell maturation by regulating the translation of the androgen receptor in an m6A-dependent manner. In addition, we identified two functional mutations of *FTO* in male infertility patients, resulting in truncated FTO protein and increased m6A modification *in vitro*. Our results highlight the crucial effects of FTO on spermatogonia and Leydig cells for the long-term maintenance of spermatogenesis and expand our understanding of the function of m6A in male fertility.

Infertility, as a worldwide problem, affects 8 to 12% of couples who have unprotected sexual intercourse at childbearing age (1). Approximately 50% of infertility cases are attributed to male factors (2, 3). N6-methyladenosine (m6A), a reversible modification of different types of RNA (4, 5), is abundant in the testis (6). Several studies have implicated the pivotal functions of m6A in male germline development and spermatogenesis through genetic ablation in animals (7, 8). Mouse models lacking methyltransferase-like 3 (*Mettl3*) and *Mettl14* specifically in male germ cells reveal that m6A plays an essential role in regulating spermatogonial stem cell maintenance and differentiation (9, 10). Mutant mice with loss of YTH domain containing 2 (*Ythdc2*) also show male infertility through alterations in gene expression involved in the transition from mitosis to meiosis and telomere clustering in pachytene cells (11, 12). In spermatocytes and round spermatids, AlkB homolog 5 (*Alkbh5*) knockout (KO) results in shorter transcripts with elevated m6A levels, suggesting the critical function of ALKBH5 in correct splicing of longer 3′-UTR transcripts (13, 14).

Fat mass and obesity-associated factor (FTO), a critical m6A demethylase (15), is expressed ubiquitously in mammalian tissues and is implicated in various physiological and biological processes (16). It has been shown in animal models that overactivation of *Fto* induces increased calorie intake and obesity (17, 18), whereas *Fto* deficiency causes thinness and growth retardation (19, 20, 21). Additionally, previous evidence has revealed the functional importance of the FTO-dependent m6A epitranscriptome in cardiac function during heart failure and postnatal neurodevelopment (22, 23). Recent research has uncovered that FTO is relevant in mouse embryonic and oocyte development by regulating the RNA abundance of long-interspersed element-1 (LINE1) and shaping the local chromatin state (24). Interestingly, it has been reported that deletion of *Fto* triggers cell cycle arrest and abnormal chromosome segregation in the mouse GC-1 spermatogonial cell line (25, 26). However, FTO has not yet been well defined *in vivo* for its role in male germline development and male fertility, particularly as an RNA demethylase.

In this study, we revealed that *Fto* depletion in mice impaired the proliferation ability of undifferentiated spermatogonia and accelerated male germ cell apoptosis, leading to spermatogenesis defects in an age-dependent manner. Mechanistically, we exposed that FTO played an m6A-dependent role in regulating protein translation of androgen receptor (AR) in Leydig cells. In addition, we identified two functional mutations of *FTO* in oligospermia and nonobstructive azoospermia (NOA) patients. Our research indicates a different role of FTO in the male reproductive system, which suggests a potential strategy for treating male infertility.

## Results

### Loss of *Fto* in mice results in spermatogenesis defects in an age-dependent manner

**Figure 1:**
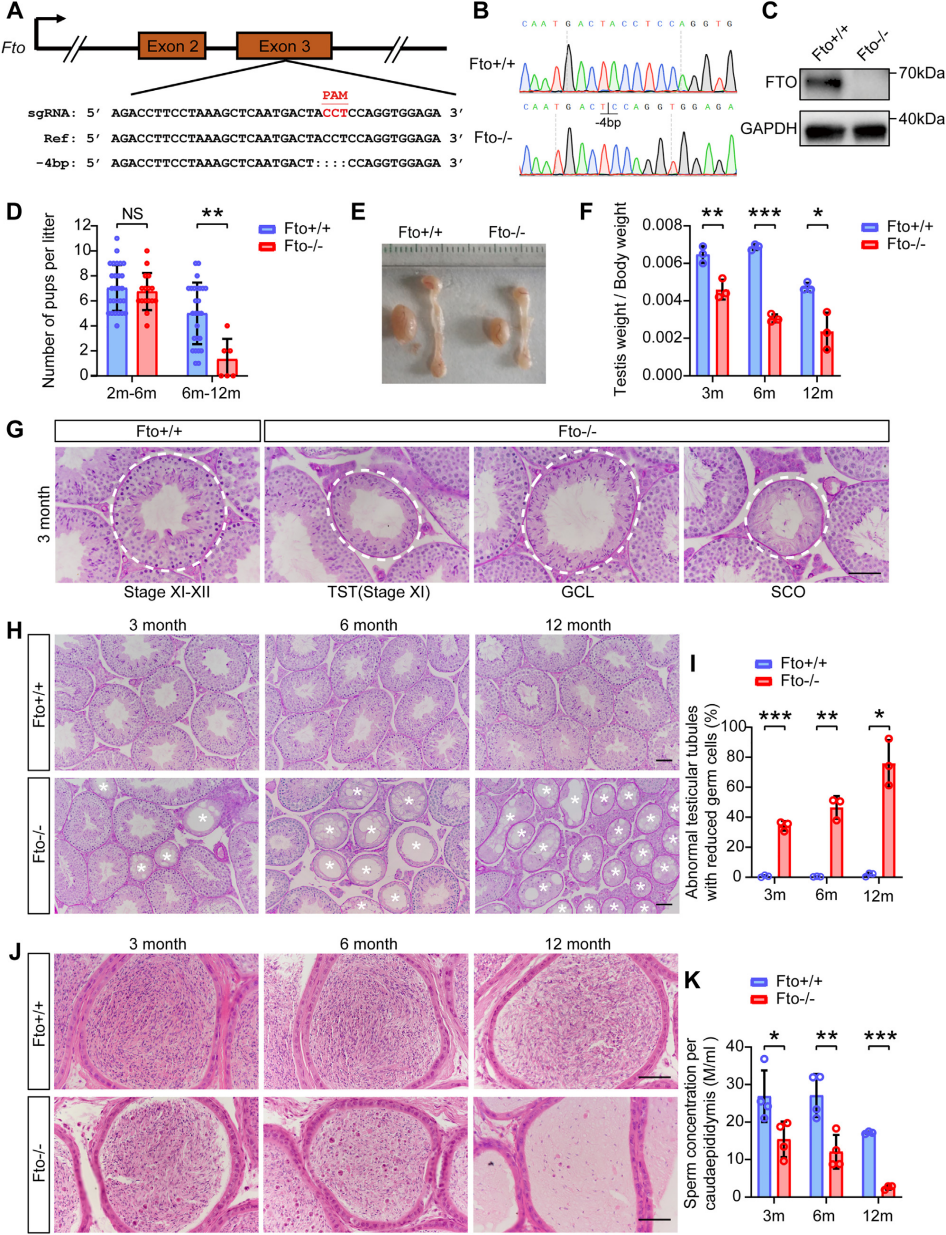
***Fto* knockout (KO) mice exhibited an age-dependent defect in spermatogenesis.***A* and *B*, schematic strategy for constructing the *Fto* KO mouse model and the result of Sanger sequencing verification. *C*, Western blot analysis of FTO from the testes of wildtype (WT) and *Fto* KO mice. GAPDH was used as a loading control. *D*, fertility analysis of the number of pups per litter for WT and *Fto* KO male mice from 2- to 6-months-old and from 6-to 12-months-old (n = 4). Bar graphs represent the means ± SDs. NS, not significant. ∗∗*p* < 0.01. *E* and *F*, the morphology and testis weight of testes and epididymides from 3-, 6-, and 12-month-old WT and *Fto* KO male mice (n = 3). Bar graphs represent the means ± SDs. ∗*p* < 0.05, ∗∗*p* < 0.01, ∗∗∗*p* < 0.001. *G*, representative images of periodic acid-Schiff (PAS)-stained testis sections showing abnormal seminiferous tubules in 3-month-old WT and *Fto* KO mice, including thinning of seminiferous tubules (TST), tubules with massive germ cell loss (GCL), and Sertoli cell only tubules (SCO). Scale bars, 50 μm. *H*–*I*, PAS staining of testis sections from 3-, 6-, and 12-month-old WT and *Fto* KO male mice (n = 3). Scale bars, 50 μm. The *white asterisks* indicate abnormal seminiferous tubules. Bar graphs represent the means ± SDs. ∗*p* < 0.05, ∗∗*p* < 0.01, ∗∗∗*p* < 0.001. *J* and *K*, hematoxylin and eosin staining and sperm concentration of cauda epididymides from 3-, 6-, and 12-month-old WT and *Fto* KO male mice (n = 4). Scale bars, 50 μm. Bar graphs represent the means ± SDs. ∗*p* < 0.05, ∗∗*p* < 0.01, ∗∗∗*p* < 0.001. FTO, fat mass and obesity-associated factor.

Quantitative real-time PCR assays showed that *Fto* mRNA displayed high expression levels in the testis and ovary (Fig. S1*A*). To examine the function of FTO in the reproductive system, an *Fto* KO mouse model was generated using CRISPR/Cas9-mediated genome editing techniques (Fig. 1, *A* and *B*). Western blotting demonstrated deletion of the FTO protein in *Fto* KO mouse testes (Fig. 1*C*). We found that *Fto* heterozygous KO mice produced a low percentage of homozygous KO offspring (Fig. S1*B*), implying homozygous lethality caused by *Fto* deletion. To test male fertility, 2-month-old wildtype (WT) and *Fto* KO male mice were cohabited with WT female mice for 10 months, and the numbers of pups per litter generated by mating of 2- to 6-month-old and 6- to 12-month-old male mice were recorded. *Fto* KO male mice were fertile at a young age but displayed reduced fertility in an age-dependent manner. Among four *Fto* KO male mice, three fathered no offspring at the age of 6 to 12 months (Fig. 1*D*). Accordingly, we also found that the ratio of testis-to-body weight of older *Fto* KO mice showed a marked reduction (Fig. 1, *E* and *F*). Testicular histology analysis of 3-month-old *Fto* KO mice exhibited a higher proportion of abnormal testicular tubules, including thinning of seminiferous tubules, tubules with massive loss of germ cells of multiple stages, and tubules with Sertoli cells only (Fig. 1*G*). Furthermore, compared with 3-month-old *Fto* KO mice, 6-month-old *Fto* KO mice had more abnormal seminiferous tubules, and up to 80% of seminiferous tubules in *Fto* KO mice were abnormal at 12 months of age (Fig. 1, *H* and *I*). Consistently, a significant reduction in sperm concentration was observed from the caudal epididymis of *Fto* KO mice compared with their WT littermates, accompanied by an extremely low sperm concentration in 12-month-old *Fto* KO mice (Fig. 1, *J* and *K*). However, the sperm motility and sperm progression rate were similar between the two groups (Fig. S2, *A* and *B*). We also found that the level of serum testosterone significantly declined in *Fto* KO mice, while the levels of serum follicle-stimulating hormone (FSH) and luteinizing hormone (LH) did not change (Fig. S2, *C*–*E*). The analysis of serum sex hormone levels suggested a nonnegligible influence of FTO on the gonadal axis. Overall, these results reveal that the male reproductive defects in *Fto* KO mice worsen with increasing age, indicating an indispensable role of FTO in the maintenance of spermatogenesis in adulthood.

Meanwhile, we found that *Fto* KO female mice were fertile but were incapable of producing healthy surviving offspring. The ovary-to-body weight ratio of 6-month-old *Fto* KO female mice was reduced by 50%, resulting in significantly smaller ovaries (Fig. S3, *A* and *B*). Histological sections with hematoxylin and eosin staining of ovaries showed that there were fewer follicles in *Fto* KO ovaries (Fig. S3*C*). Moreover, ELISAs displayed a reduction in serum estradiol (E2) and anti-Mullerian hormone (AMH) levels and a rise in serum FSH levels in *Fto* KO female mice (Fig. S3, *D*–*F*). Therefore, FTO is also required for folliculogenesis, and its deletion might lead to premature ovarian failure.

### *Fto* deletion causes defective spermatogonial cells

**Figure 2:**
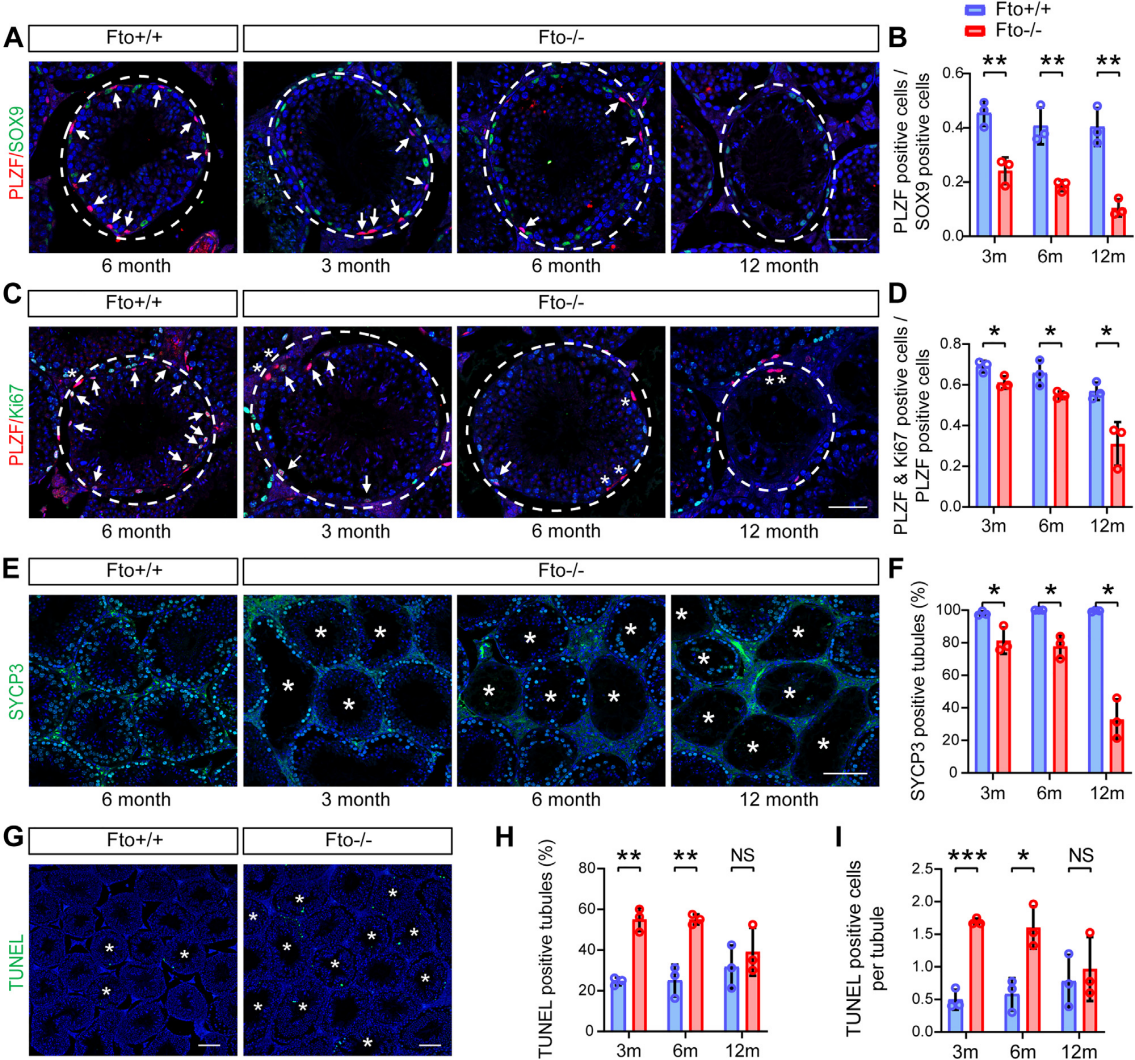
***Fto* KO testes showed reduced proliferation of undifferentiated spermatogonia and increased male germ cell apoptosis.***A*, immunostaining for promyelocytic leukemia zinc-finger (PLZF)-positive cells (*red*, undifferentiated spermatogonia) and sex determining region Y-Box 9 (SOX9)-positive cells (*green*, Sertoli cells) per spermatogenic tubule in 3-, 6- and 12-month-old WT and *Fto* KO male mice. The *white arrows* indicate PLZF-positive cells. Scale bars, 50 μm. *B*, the ratios between undifferentiated spermatogonia and Sertoli cells per seminiferous tubule in testis sections from 3-, 6-, and 12-month-old WT and *Fto* KO male mice (mice, n = 3; tubules of one mouse, n = 20). Bar graphs represent the means ± SDs, ∗∗*p* < 0.01. *C*, representative seminiferous tubules of PLZF (*red*) immunostaining and Ki67 (*green*) immunostaining from 3-, 6-, and 12-month-old WT and *Fto* KO male mice. The *white arrows* indicate PLZF and Ki67 double-positive cells, and *white asterisks* indicate PLZF single-positive cells. Scale bars, 50 μm. *D*, the proportions of proliferating undifferentiated spermatogonia (PLZF & Ki67 positive) among all undifferentiated spermatogonia (PLZF positive) from 3-, 6-, and 12-month-old WT and *Fto* KO male mice (mice, n = 3; tubules of one mouse, n = 20). Bar graphs represent the means ± SD. ∗*p* < 0.05. *E*, images of testis sections with immunostaining for spermatocyte marker synaptonemal complex protein 3 (SYCP3) (*green*) from 3-, 6- and 12-month-old WT and *Fto* KO male mice. The *white asterisks* show defective tubules. Scale bars, 100 μm. *F*, the percentages of SYCP3-positive seminiferous tubules in 3-, 6-, and 12-month-old WT and *Fto* KO male mice (n = 3). Bar graphs represent the means ± SDs. ∗*p* < 0.05. *G*, images of TUNEL staining of testicular sections from 6-month-old WT and *Fto* KO male mice. The *white asterisks* indicate TUNEL-positive tubules. Scale bars, 100 μm. *H* and *I*, frequencies of TUNEL-positive tubules (*H*) and average numbers of TUNEL-positive cells per tubule (*I*) in 3-, 6- and 12-month-old WT and *Fto* KO male mice (mice, n = 3; tubules of one mouse, n = 80). Bar graphs represent the means ± SDs. NS, not significant, ∗*p* < 0.05, ∗∗*p* < 0.01, ∗∗∗*p* < 0.001. FTO, fat mass and obesity-associated factor.

To clarify the function of FTO in male germ cells, we analyzed germ cell properties in testes from 3-, 6- and 12-month-old WT and *Fto* KO male mice. First, immunofluorescence staining for the Sertoli cell marker sex determining region Y-Box 9 (SOX9) in testes indicated that the SOX9-positive cell numbers were unchanged in *Fto* KO mice of all ages (Figs. 2*A* and S2*F*). We next performed immunostaining of testicular sections for promyelocytic leukemia zinc-finger (PLZF), a common marker for undifferentiated spermatogonia. Interestingly, the average ratio of PLZF-positive cells to SOX9-positive cells was significantly reduced by loss of *Fto* in male germ cells in an age-dependent manner (Fig. 2, *A* and *B*). To determine whether FTO deficiency impaired the proliferation of undifferentiated spermatogonia, we compared the proliferation among the PLZF-positive undifferentiated spermatogonia by double immunostaining for the mitosis marker Ki67. As expected, the ratio of PLZF- and Ki67-positive cells to PLZF-positive cells in *Fto* KO testes declined compared with that in the control group (Fig. 2, *C* and *D*). Subsequently, the number of synaptonemal complex protein 3 (SYCP3)-positive meiotic germ cells was also reduced in *Fto* KO testes (Fig. 2, *E* and *F*). However, the comparison of WT and *Fto* KO mice revealed no difference in the ratio of stimulated by retinoic acid 8 (STRA8)-positive cells (a differentiated spermatogonia marker) to PLZF-positive cells, indicating that spermatogonial differentiation was not affected by loss of *Fto* (Fig. S2, *G* and *H*). In addition, the TUNEL assay showed significantly increased germ cell apoptosis in 3- and 6-month-old *Fto* KO mice compared with WT mice, but there was no difference at 12 months of age (Fig. 2, *G*–*I*). These observations suggest that deletion of *Fto* reduces the proliferation ability of undifferentiated spermatogonia and induces male germ cell apoptosis during spermatogenesis.

### Detection of the m6A-dependent regulation of AR by FTO

**Figure 3:**
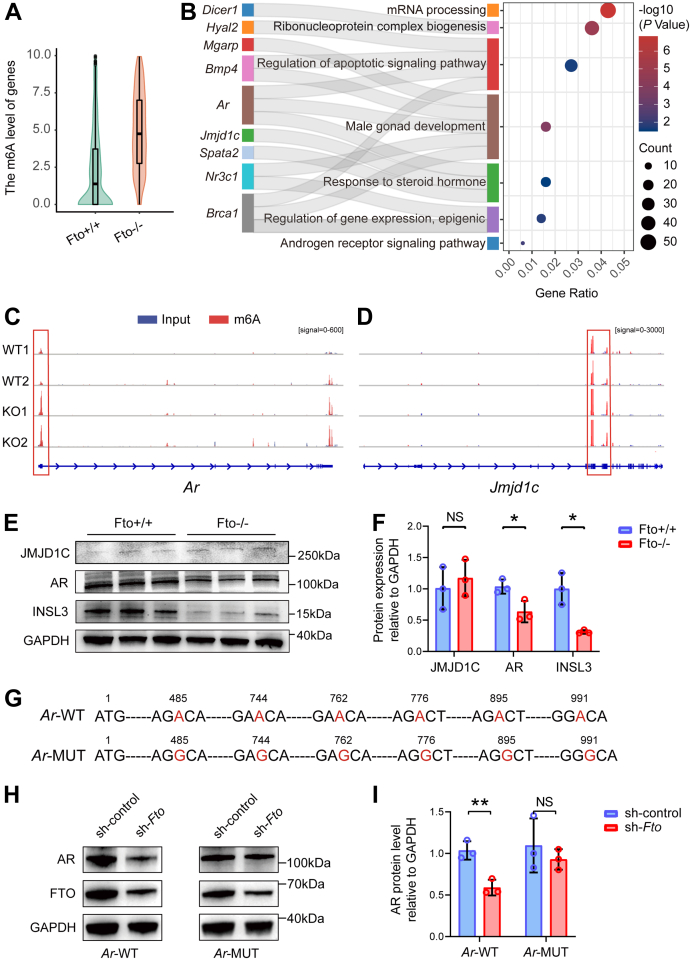
**Detection of the m6A-dependent regulation of AR by FTO.***A*, violin plot of the m6A level of protein-coding genes from 6-month-old WT and *Fto* KO mouse testes. The *p* value was adjusted using the Wilcoxon rank test (*p* = 4.14 × 10^−176^). *B*, Gene Ontology enrichment analysis of 1719 genes in which the m6A levels within exons in the *Fto* KO mouse group were higher than those in the WT mouse group. *p* values were adjusted using the Benjamini‒Hochberg procedure. *C* and *D*, IGV software analysis of the m6A peaks of *Ar* and *Jmjd1c* mRNA transcripts in 6-month-old WT and *Fto* KO mouse testes. The *red bars* are m6A-modified peaks, and the *blue bars* are input peaks. Signal represents the abundance of input or m6A. The m6A abundances is assessed by the difference between the signals of m6A-modified peaks and input peaks. *E* and *F*, Western blot and quantitative analysis of JMJD1C, AR, and INSL3 from the testes of 6-month-old WT and *Fto* KO mice. GAPDH was used as a loading control (n = 3). Bar graphs represent the means ± SDs. NS, not significant, ∗*p* < 0.05. *G*, schematic representation of mutations in pcDNA3.1-*Ar*-CDS is shown. *H*–*I*, TM3 cells were transfected with sh-*Fto* followed by transfection with pcDNA3.1-*Ar*-WT and mutant (MUT) plasmids, and cell lysates were subjected to Western blotting and quantitative analysis. Bar graphs represent the means ± SDs. NS, not significant, ∗∗*p* < 0.01. AR, androgen receptor; FTO, fat mass and obesity-associated factor.

To explore the possible mechanisms by which FTO functions during spermatogenesis, we first examined the location of FTO protein within seminiferous tubules. Immunohistochemical and immunofluorescence assays showed that FTO was expressed in Leydig cells and PLZF-positive undifferentiated spermatogonia (Fig. S4, *A* and *B*). As FTO is a known critical m6A demethylase, we performed m6A-MeRIP sequencing using testes from WT and *Fto* KO mice (Fig. S5*A*). m6A-MeRIP sequencing analysis identified 19,127 and 37,760 m6A peaks, including 6158 and 8566 m6A-modified genes in the WT and *Fto* KO groups, respectively. Consistent with previous studies, m6A peaks were abundant in the CDS and the 3′UTR (Fig. S5*B*). The correlation analysis of the m6A levels showed that the intragroup samples clustered well according to their counterpart group after gene screening (Fig. S5*C*). Furthermore, the m6A levels of these genes in the *Fto* KO group were significantly higher than those in the WT group, demonstrating increased m6A demethylation caused by the deletion of *Fto* in the testis (Fig. 3*A*). However, compared with the WT group, the proportion of m6A peaks in exons showed a reduction (35.8% to 24.0%) in the *Fto* KO group through whole transcriptome analysis (Fig. S5*D*). Next, we performed Gene Ontology (GO) enrichment analysis on the genes with increased m6A levels in exons from the *Fto* KO group. We noticed that these genes were clustered in the GO terms of male gonad development, response to steroid hormone and AR signaling pathway (Fig. 3*B*). Then, we performed literature searching and consulted the function of genes with differential m6A methylation levels in the GO analysis pathways to screen potential downstream targets of FTO. We screened genes that played important functions in spermatogenesis, including *Ar*, *Jmjd1c*, *Bmp4*, *Brca1*, *Dicer1*, *Hyal2*, *Nr3c1*, *Spata2*, and *Mgarp*. The abundance of m6A peaks on the transcripts of these genes was significantly higher in *Fto* KO mice than in WT mice as verified by IGV (Figs. 3, *C* and *D* and S6, *A*–*G*). Considering that FTO is expressed in undifferentiated spermatogonia and Leydig cells, JMJD1C localized in undifferentiated spermatogonia (27) and AR localized in Leydig cells (28) were further screened from the above genes. Western blot analysis revealed that the protein level of AR, but not JMJD1C, was significantly reduced in *Fto* KO mice (Fig. 3, *E* and *F*). Given that autocrine AR action is of great importance to the maturation of Leydig cells and the maintenance of spermatogenesis in adult mice (29, 30), we examined whether the expression of insulin-like 3 (INSL3), a marker of Leydig cell maturation, was affected. We found that the protein level of INSL3 was also markedly lower in *Fto* KO group than in WT group (Fig. 3, *E* and *F*), while the transcript of *Insl3* did not have elevated m6A modification in *Fto* KO group (Fig. S6*H*). It indicated that *Insl3* was not a direct target of FTO.

To further verify the m6A-modified regulatory impact on AR, we knocked down *Fto* using a lentiviral vector expressing *Fto*-specific shRNA, accompanied by the transfection of *Ar* overexpression plasmids containing WT cDNA construct and its mutant (MUT) counterpart in a mouse TM3 Leydig cell line. The m6A motifs in exon one of *Ar* identified by m6A-MeRIP sequencing of testes were mutated from A to G in the MUT overexpression plasmid (Fig. 3*G*). Similar to the result in mouse testis, we observed a markedly reduced protein level of AR after the transfection of *Fto* shRNA and *Ar*-WT plasmids. However, AR expression in TM3 cells overexpressing the *Ar*-MUT plasmid was not significantly changed under knockdown of *Fto* (Fig. 3, *H* and *I*). These results indicate that FTO regulates Leydig cell maturation and maintenance of spermatogenesis through AR in an m6A-dependent manner.

### Identification of deleterious *FTO* mutations in patients with oligospermia and NOA

**Figure 4:**
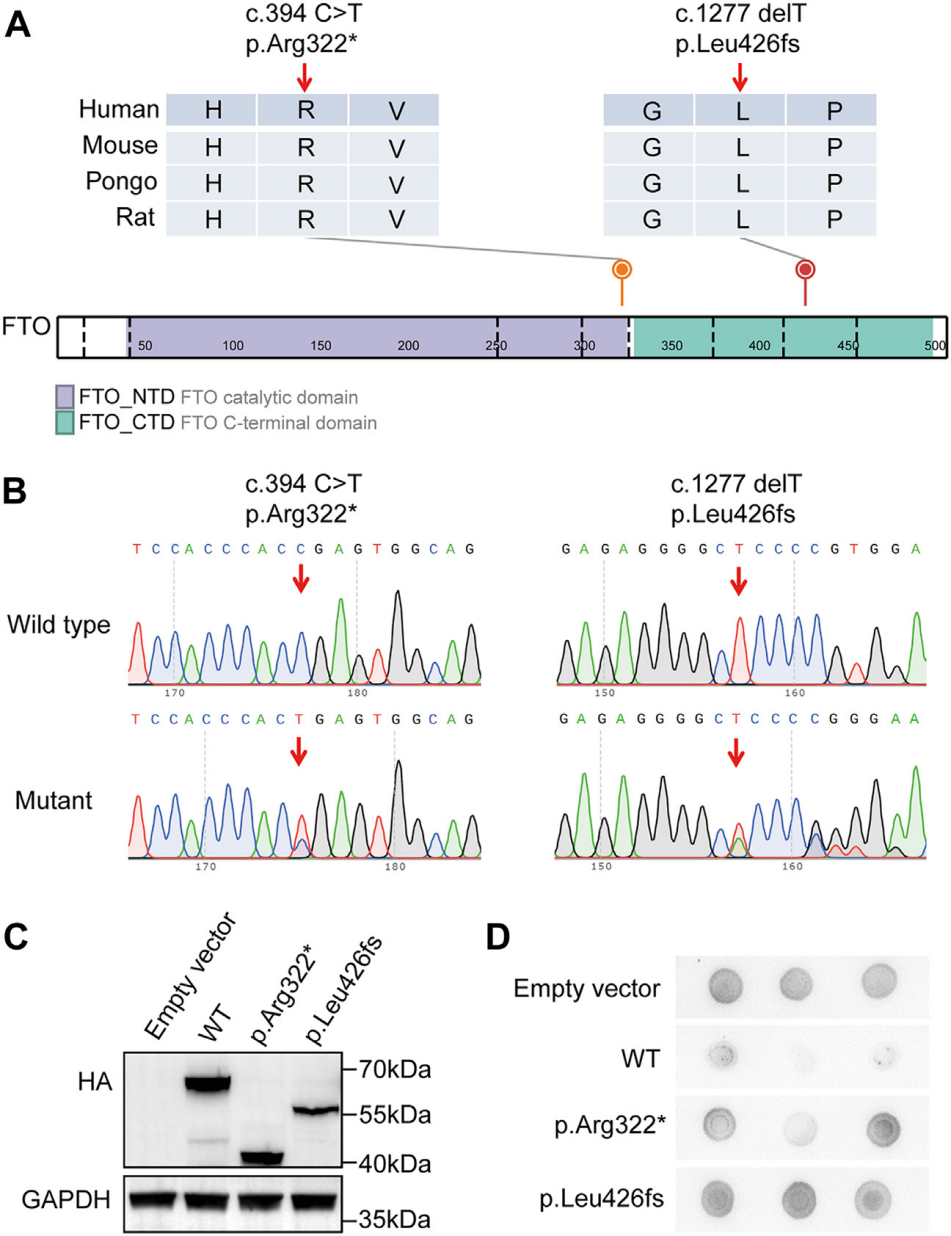
**Identification of deleterious *FTO* mutations in infertile males.***A*, schematic representation of the domains of FTO and locations of *FTO* variants identified in this study. Sequence alignment shows conservation of the mutated residues across different species according to the UCSC genome browser. The *purple box* indicates the FTO catalytic domain, and the *green box* indicates the FTO C-terminal domain as described by the UniProt server. *B*, Sanger sequencing graphs of two mutations identified from patients with oligospermia and nonobstructive azoospermia. *C*, Western blot assays of the overexpression of *Fto* with two mutations in HEK293T cells transfected with pcDNA3.1-*Fto*-CDS and mutant plasmids containing the HA tag sequence. GAPDH was used as a loading control. *D*, m6A dot blot analysis of mutant forms of *Fto* in HEK293T cells. CTD, C-terminal domain; FTO, fat mass and obesity-associated factor; NTD, N-terminal domain.

According to the UCSC genome browser and the UniProt server, DNA and amino acid sequence of FTO in mouse are highly homologous to those in human (Fig. S7). To evaluate the potential function of FTO in human fertility, whole-exome sequencing (WES) data for patients with oligospermia and NOA (total 1001) were utilized to screen for loss-of-function mutations in *FTO*. We identified a heterozygous nonsense mutation (NP_001073901: c.964C>T, p. Arg322∗) in patients with NOA and a heterozygous frameshift mutation (NP_001073901: c.1277delT, p. Leu426fs) in patients with oligospermia. Two mutations were absent in the human genome datasets archived in the ExAC and gnomAD databases and were located in the FTO catalytic domain and C-terminal domain (Table 1, Fig. 4*A*). We then confirmed two mutations through Sanger sequencing (Fig. 4*B*). To further evaluate the impacts of the above mutations *in vitro*, WT and MUT cDNA constructs of *FTO* with HA tags were overexpressed in the HEK293T cell line. Western blot assays displayed significant truncation of FTO protein in both MUT cDNA constructs, accompanied by decreased protein levels in the frameshift MUT construct (Fig. 4*C*). Moreover, the above two mutations resulted in an increase in global m6A levels as determined by m6A dot blot assays (Fig. 4*D*). These results suggest that two functional mutations associated with oligospermia and NOA play important regulatory roles in the protein expression of FTO and m6A modification.

**Table 1:** Characteristics of two *FTO* variants identified in infertility males

Characteristics	Patient 1	Patient 2
Variant coordinate	Chr16: 53907766	Chr16: 53967934
cDNA alteration	c.964C>T	c.1277delT
Amino-acid variation	p. Arg322∗	p. Leu426fs
Variant type	Nonsense	Frameshift
Maf_case^a^	0.000499	0.000499
Maf_ExAC (all/Asian)	0/0	0/0
Maf_gnomAD (all/Asian)	0/0	0/0
CADD^b^	44	NA
Variant allele	Heterozygous	Heterozygous

Abbreviation: NA, not available.

a N_Case_= 1001.

b The function of mutations are predicted by Combined Annotation Dependent Depletion (CADD) tool.

## Discussion

Our research showed that deletion of *Fto* in mice led to spermatogenesis defects in an age-dependent manner. Furthermore, we observed that FTO was significantly involved in Leydig cell maturation and maintenance of spermatogenesis by regulating the protein level of AR in an m6A-dependent manner. We also identified two functional variants of *FTO* in oligospermia and NOA patients, which had a potential pathogenic effect on male infertility.

The demethylase FTO plays a crucial role in removing the m6A modifications in RNA(4). FTO has been reported to be associated with premature ovarian insufficiency disease (31). Furthermore, *Fto* KO female mice show a decreased number of germinal vesicle oocytes and damaged maturation to the mature MII oocyte stage, accompanied by increased chromosome misalignment and spindle collapse regulated by LINE1 (24). A previous study showed that deletion of *Fto* did not result in obvious spermatogenesis defects in mice (19). In our research, we found that *Fto* KO male mice were fertile at a young age while exhibiting reduced fertility in an age-dependent manner, accompanied by a gradually reduced ratio of testis-to-body weight and an increasing number of abnormal seminiferous tubules. The reason for the different observations might be the lack of investigation for the sustainability of male fertility over time in those previous studies. Subsequently, we investigated the cause for the age-dependent decrease in the fertility of *Fto* KO male mice. We observed that *Fto* depletion led to a reduced proliferation ability of undifferentiated spermatogonia. Given that FTO protein was expressed in spermatogonia and Leydig cells, we originally considered to apply sorted spermatogonia and Leydig cells for m6A-MeRIP sequencing to further explore the possible mechanisms. However, we found that *Fto* heterozygous KO mice produced a low percentage of homozygous KO offspring, and *Fto* homozygous KO female mice were incapable of producing surviving offspring, which was consistent with previous findings (24). Therefore, we were unable to obtain enough 7-day-old homozygous KO male mice to separate spermatogonia and Leydig cells for m6A-MeRIP sequencing. Consequently, total RNA of testis extracts from adult WT and *Fto* KO male mice was employed for the subsequent experiments. After GO enrichment analysis and literature searching, we focused on *Ar* as a potential target under m6A-dependent regulation by FTO for subsequent analysis.

AR, an essential receptor for testosterone, is expressed in Leydig, peritubular myoid, Sertoli, vascular smooth muscle, and vascular endothelial cells of the mature testis (28). AR plays different functions in different types of male germ cells (30). A total *Ar* KO mouse model is entirely infertile and has small testes. Some tubules of *Ar* KO mice only have a few spermatogonia, while the others are completely devoid of germ cells (32, 33). Leydig cell-specific *Ar* KO mice (ARLCKO) are fertile but exhibit seminiferous epithelium degeneration with aging and decreased maturation of Leydig cells with low expression of INSL3 (29). It has been reported that INSL3, a Leydig cell maturation marker, protects against germ cell loss or apoptosis (34). In our research, similar to ARLCKO mice, *Fto* KO mice were fertile at a young age and displayed age-dependent spermatogenesis defects, increased male germ cell apoptosis, and decreased INSL3 protein expression in testes. However, the difference in cell apoptosis was more obvious between young WT and *Fto* KO mice but was nonsignificant when the mice were 12-month-old. The reasons may include two points: (1) In normal mouse testis, testicular germ cells underwent physiological apoptosis. It was reported that the proportion of apoptosis in meiotic spermatogenic cells increased with age, which was a result of natural senescence of testis (35, 36, 37); (2) As loss of *Fto* reduced the proliferation ability of undifferentiated spermatogonia and induced male germ cell apoptosis during spermatogenesis, more than 80% of the seminiferous tubules of 12-month-old *Fto* KO mice showed Sertoli cell-only, companied by a significantly reduce in spermatogenic cells. Therefore, less TUNEL signals were detected in the tubules with massive germ cell loss of 12-month-old *Fto* KO mice than that of 3- and 6-month-old *Fto* KO mice. In addition to the protein localization of FTO in Leydig cells, we also found higher m6A levels and lower protein levels of AR caused by FTO deficiency. We further demonstrated FTO-mediated m6A modifications on AR in the TM3 Leydig cell line. Consequently, these phenotypes of *Fto* KO male mice could be explained by insufficient AR. However, different from the unchanged testosterone level of ARLCKO mice, the serum testosterone level was significantly decreased in *Fto* KO male mice, accompanied by unchanged serum FSH and LH levels. It indicated that reduced testosterone level of *Fto* KO mice did not induce the feedback of increased FSH and LH levels. Testosterone is essential for male gonad development and adult spermatogenesis by controlling the expression of various genes in different types of somatic cells in the testis (30). Some of these genes then act on spermatogonial stem cells. Glial cell derived neurotrophic factor secretion is regulated by endocrine peritubular myoid cells *via* testosterone synthesized by Leydig cells to stimulate spermatogonial stem cell proliferation (38). Therefore, the analysis of serum sex hormone levels suggested a damaged hypothalamic–pituitary–gonadal axis in *Fto* KO male mice, which might be independent of the regulation by AR. In addition, recent studies have demonstrated that m6A modifications are functionally involved in controlling mouse embryonic stem cell fate and early embryonic development (39, 40, 41). Deficiency of *Mettl3*, *Ythdc1*, and *Fto* in mice results in early embryonic lethality (19, 42, 43). LINE1 RNA was identified as a key target of METTL3, YTHDC1, and FTO. After *Mettl3* depletion in mouse embryonic stem cells, reduced m6A methylation multiplies the levels of carRNAs (mainly LINE1 repeats) and facilitates chromatin openness and downstream transcription (44). Increased m6A on LINE1 RNA caused by *Fto* KO could promote YTHDC1 binding, which restrains the chromatin accessibility and transcription of early development and pluripotency genes (24). Considering the critical role of m6A in embryonic development, the function of FTO in spermatogenesis may already be evident in the embryonic period. However, additional research still needs to be performed. It is necessary to generate spermatogonia or Leydig cell-specific *Fto* KO mouse models to further confirm the spermatogenic effect of FTO.

To clarify the function of FTO in human fertility, two loss-of-function mutations in *FTO* were identified in patients with oligospermia and NOA. Two variants were absent in the human genome datasets of East Asian populations archived in the ExAC and gnomAD databases. FTO consists of 505 residues, which can be segmented into an N-terminal domain (NTD, residues 32–326) and a C-terminal domain (CTD, residues 327–498). A distorted double stranded β-helix construct constitutes the main catalytic core of the NTD for RNA demethylase activity (45). The CTD and the NTD can interact to diminish the construction of the NTD; hence, the CTD domain also significantly contributes to the activation of demethylation activity (46). The nonsense mutation (NP_001073901: c. 964C>T, p. Arg322∗) and the frameshift mutation (NP_001073901: c. 1277delT, p. Leu426fs) identified in this study are located in the NTD and CTD, respectively. We found that two variants both resulted in truncation of the FTO protein and elevated m6A levels *in vitro*, leading to damage to the demethylation activity of FTO. In consideration of age-dependent spermatogenic defects caused by deletion of *Fto*, we checked the age at which two patients were diagnosed. Interestingly, we found that the patient with oligospermia was 26 years old; however, the patient with NOA was 37 years old, which is in accordance with the finding that older *Fto* KO male mice suffered more severe spermatogenic disorders (Table S1). These findings imply that agonists of FTO in the testis might have potential clinical value for treating oligospermia or NOA, especially for middle-aged male patients. Nevertheless, because activation of FTO might trigger negative effects in cancers (47, 48), further well-established studies are needed to validate the potential therapeutic effect of FTO on male infertility.

In conclusion, we identified two loss-of-function mutations in *FTO* from patients with oligospermia and NOA and revealed the critical role of FTO in modulating the proliferative ability of undifferentiated spermatogonia and Leydig cell maturation in mice through the regulation of AR in an m6A-dependent manner. Our research expands the understanding of the role of m6A modification in regulating the long-term maintenance of spermatogenesis, which is expected to provide new strategies for the treatment of infertile males by using agonists of FTO in the testis.

## Experimental procedures

### Antibody

The following antibodies were employed for immunostaining or Western blotting: rabbit polyclonal anti-FTO (Proteintech, 27226-1-AP, for Western blotting 1:3000, for immunofluorescence or immunohistochemistry 1:200), rabbit polyclonal anti-SOX9 (Millipore, AB5535, for immunofluorescence 1:400), goat polyclonal anti-PLZF (R&D, AF2944, for immunofluorescence 1:200), rat monoclonal anti-Ki67 (Invitrogen, 14-5698-95, for immunofluorescence 1:500), mouse monoclonal anti-SYCP3 (Abcam, ab97672, for immunofluorescence 1:500), rabbit polyclonal anti-STRA8 (Abcam, ab49602, for immunofluorescence 1:200), rabbit polyclonal anti-AR (Abcam, ab74272, for Western blotting 1:100), rabbit polyclonal anti-JMJD1C (ABclone, A20153, for Western blotting 1:500), and rabbit polyclonal anti-INSL3 (ABclone, A5728, for Western blotting 1:500).

### Clinical sample information

In this research, oligospermia and NOA cases (total 1001) were enrolled from the Reproductive and Genetic Hospital of CITIC-Xiangya (Changsha, China). All male recruits were Han Chinese men who were genetically unrelated to each other and were selected after undergoing an andrological examination, including history and physical examination, scrotal ultrasound, hormone analysis, semen analysis, karyotyping and Y chromosome microdeletion screening. Exclusion criteria for the study included those with a history of vasectomy, vascular trauma, cryptorchidism, obstruction of the vas deferens, orchitis, abnormalities in chromosome number, or microdeletions of the oligospermia and NOA factors region on the Y chromosome. Sperm could be detected in the ejaculate of subjects with oligospermia, but the total number of sperm in one ejaculation was less than 39 × 10^6^ (or the sperm concentration was less than 15 × 10^6^/ml) after at least two routine semen analyses. Subjects with NOA were diagnosed by the results of at least three semen analyses, in which no sperm were found by centrifugation. Semen analysis was carried out under World Health Organization criteria (2010). Before taking part in this study, all patients provided written informed consent, and each of them provided a 5 ml sample of whole blood to extract genomic DNA for further sequencing analysis.

### WES and screening of variants in the *FTO* gene

Genomic DNA was extracted using a whole blood DNA purification kit (Qiagen). The processes of WES and bioinformatics analysis were performed as previously described (49, 50). In brief, an Illumina HiSeq 2000 sequencer was utilized for the sequencing of genomic DNA sequencing libraries, which were prepared by the Agilent Sure-Select Human All Exon V6 Kit. Raw data reads were aligned to the human reference genome (hg19, GRCh37) by the Burrows‒Wheeler Aligner. Then, we conducted functional annotation using ANNOVAR software through various open-access databases, such as gnomAD and ExAC. Frameshift and nonsense variants were identified as candidate variants, which should be simultaneously absent in public population genome databases. Further validation of *FTO* variants identified by WES was performed *via* Sanger sequencing with the primers listed in Table S2.

### Mouse model and cell line

To generate *Fto* KO mice, single-guide RNA (sgRNA) was designed to target exon three of *Fto*. The sgRNA expression plasmid was generated using oligonucleotides, which were then annealed and cloned into the pGL3-U6-sgRNA-PGK-puromycin expression vector (Addgene, 51133). CRISPR/Cas9 plasmids were transcribed and microinjected *in vitro*. In brief, BsaI was utilized to linearize pGL3-T7-sgRNA-PGK-puromycin expression vectors, which were then subjected to transcription *in vitro* using the MEGA short script Kit (Ambion). Linearization and transcription of the Cas9 plasmid (pST1374-NLS-flag-linker-Cas9, Addgene 44758) were carried out using the AgeI and T7 Ultra Kit (Invitrogen). The cytoplasm and male pronucleus of the zygote were electroporated with the injection of a mixture of sgRNA and Cas9 mRNA. According to standard procedures, embryos were transplanted into C57BL/6J females who were feigning pregnancy. C57BL/6J mice were backcrossed with founder mice. PCR amplification and Sanger sequencing confirmed a 4 bp deletion in *Fto* homozygous KO mice and did not detect any predicted off-target sites (Fig. S1*C*). All mice were housed in a specific pathogen-free animal facility under standard conditions. All animal experiments in this study were approved by the Institutional Animal Care and Use Committee of Nanjing Medical University, Nanjing, China. Table S3 contains a list of the primers used for the genotyping identification of *Fto* KO mice. Table S4 contains a list of the primers used for off-target analysis.

The human HEK-293T cell line and the mouse TM3 Leydig cell line were utilized in experiments for this study.

### Construction of plasmid and shRNA

Gene sequences coding for mouse *Ar* were cloned into the pcDNA3.1 vector, and human *FTO* were cloned into the pcDNA3.1 vector containing the sequence of HA tag. The Mut Express MultiS Fast Mutagenesis Kit (Vazyme, C215-02) was used to construct point MUT plasmids *in vitro*. Lentiviral vector expressing *Fto*-specific shRNA was designed and constructed by Tsingke. The sequence for *Fto*-specific shRNA was 5′-CCGGGATGATGAAGTGGACCTTAAGCTCGAGCTTAAGGTCCACTTCATCATCTTTTTG-3′.

### Quantitative real-time PCR assays

Total RNA was extracted from mouse tissues using TRIzol reagent (Invitrogen) and was reverse transcribed into cDNA by PrimeScript RT Master Mix (Takara). ChamQ SYBR qPCR Master Mix (Vazyme) was utilized to carry out real-time PCR on an iCycler RT‒PCR Detection System (Bio-Rad Laboratories). Data analysis was performed using the ΔΔCT method. Each sample was repeated three times for each assay. The *Gapdh* gene was used as an internal control. Table S5 contains a list of the real-time PCR primers.

### Histological analysis

The tissues were fixed in modified Davidson fluid (5% glacial acetic acid, 15% ethanol, 30% of a 37–40% formaldehyde stock solution, and 50% distilled water). Then, the testes were embedded in paraffin for PAS staining. Sections were cut into 5-μm thick pieces, dewaxed with xylene, hydrated, PAS stained, dehydrated with an ethanol gradient (70%, 80%, 90%, and 100%), and blocked with resin. For hematoxylin and eosin staining, the epididymis and ovary sections underwent deparaffinization, hydration, staining, dehydration, and blocking.

### Immunofluorescence and immunohistochemistry analysis

For immunofluorescence, the sections were subjected to citrate buffer-mediated heat-induced antigen retrieval (8.2 mM sodium citrate, 1.8 mM citric acid, pH 6.0) and then rinsed with phosphate-buffered saline (PBS), soaked in 0.3% PBS-Triton X-100, blocked with 5% donkey serum, and incubated with primary antibodies overnight at 4 °C. After incubation with secondary antibodies and washing with PBS, the samples were sealed with Antifade Mounting Medium (Beyotime).

For immunohistochemistry, after antigen retrieval and washing with PBS, the slides were incubated with 3% H_2_O_2_ in methyl alcohol for 10 min to block endogenous peroxidase activity. Sections were then blocked with 5% donkey serum and incubated with primary antibodies overnight at 4 °C. After washing in PBS, the secondary biotinylated antibody was dripped on the tissues for a 1-h incubation. Chromogen 3,3′-diaminobenzidine was applied to tissue sections, and they were incubated for 2 min after being rinsed with PBS buffer. The slides were washed once more in PBS, stained with hematoxylin, washed with water, dehydrated with an alcohol gradient (70%, 80%, 90%, and 100%), cleared with xylene, and mounted with resin.

### TUNEL assays

An Apoptosis Detection Kit (Vazyme, A112) was utilized to conduct TUNEL assays on paraffin sections of testes from male mice. The steps were completed according to the manufacturer's instructions.

### Sperm mobility assays

Sperm from the epididymal tail were incubated in HTF medium (Irvine Scientific) containing 10% fetal bovine serum for 5 min at 37 °C. Hamilton Thorne’s Ceros II system (Beverly) was used for the measurement of sperm motility.

### Serum hormone measurements

Blood was collected from the eyes of the mice at approximately 2:00 P.M. For hormonal testing, whole blood was centrifuged at 1000 rpm for 15 min to extract plasma, which was then kept at −80 °C. The levels of serum testosterone, FSH, and LH from male mice were determined by Testosterone ELISA Kit (Arbor Assays, K032-H1), FSH ELISA Kit (Abbexa, abx154038), and LH (S-type) Rat ELISA KIT (FUJIFILM Wako, 630-23929), respectively. E2 ELISA kit (MYBioSource, MBS8800210), AHM ELISA kit (LSBio, LS-F6145-1), and FSH ELISA kit (MYBioSource, MBS2700327) were employed to measure the levels of serum E2, AMH, and FSH from female mice, respectively. An ELISA microtiter plate reader (BioTek Synergy2) was utilized to determine the absorbance at 450 to 620 nm.

### m6A-MeRIP sequencing

Total RNA was isolated by TRIzol reagent and fragmented into 100 to 200 nucleotide-long fragments using sodium acetate. Approximately 15 μg of fragmented RNA was subjected to immunoprecipitation using an m6A-specific antibody magnetic bead complex (Millipore, MABE1006). After stringent washing with low-salt IP buffer twice and high-salt buffer twice, the elution and purification of bound RNA were performed using the RNeasy MinElute Cleanup Kit (QIAGEN, 74204). Then, the purified RNA was used to construct a library using the SMARTer Stranded Total RNA-Seq Kit v2-Pico Input Mammalian Components (Takara, 634419) for Illumina. Sequencing was executed using the Illumina HiSeq 2500 platform. Ten nanograms of fragmented total RNA was constructed as input RNA using the SMARTer Stranded Total RNA-Seq Kit v2-Pico Input Mammalian Components (Takara, 634419). The Illumina HiSeq 4000 platform was used for sequencing of input RNA following the manufacturer’s instructions.

### m6A-MeRIP sequencing analysis

To carry out quality control and trimming of adaptors, raw sequencing reads were initially subjected to Trim_galore (http://www.bioinformatics.babraham.ac.uk/projects/trim_galore/). The quality threshold value was set to 20, and after trimming, the reads were at least 30 nt in length. All m6A-seq and input raw data reads were aligned against rRNA (mm10, downloaded from UCSC Genome Browser) using Bowtie2 (version 2.4.1), with the unmapped reads kept for further analysis. The remaining reads were mapped to the mm10 mouse genome using HISAT2 (version 2.2.1) with default parameters. Then, the results obtained from HISAT2 were transformed into bam format using BEDTools (version 2.29.2).

To confirm the presumptive m6A sites, the m6A-enriched sites in every sample were identified by MACS2 (version 2.2.7) with the corresponding control input sample. The effective mouse genome size was set to 1.87 × 10^9^, while the option selected was --nomodel, and the cutoff of *q* value was 0.01. We constructed the UCSC Genome Browser mm10 annotation file using the makeTxDbFromGFF function of the GenomicFeatures R package. The R package ChIPseeker was used to annotate the narrowPeak files obtained by MACS2. To calculate the m6A methylation level of genes, the peaks for which 50% of the m6A modifications were located in the gene were selected for each gene. Then, for each gene, the methylation level of each peak was multiplied by the sum of the lengths of each peak divided by the length of the longest transcript of that gene. To remove random error, the gene screening criteria for the two biological duplicate samples in the group were as follows: (1) The m6A score of any genes was greater than 1; (2) Both genes were methylated in the two biological duplicate samples in the group, and the fold change of the m6A score was less than 2. After screening, the m6A-modified genes were used for the following analysis. For data visualization, GO enrichment analysis was performed using the clusterProfiler and ggplot2 R packages. The distribution of m6A sites in mRNA was analyzed by an R package named Guitar. The heatmap of Pearson correlation and the violin plot of m6A-modified genes were analyzed by the ggplot2 R package. IGV (version 2.4.15) was employed to show the read coverage of the m6A peaks.

### m6A dot blot assays

After incubation at 95 °C for 3 min, the RNA sample was loaded onto a nitrocellulose filter membrane (Millipore) and cross-linked with UV light. The membrane was blocked with 5% nonfat dry milk for 1 h and incubated with a specific anti-m6A primary antibody (Synaptic Systems, 202003) overnight at 4 °C. Subsequently, the HRP-conjugated secondary antibody was added to the membrane at room temperature for 1 h. Finally, development was performed with Immobilon Western HRP Substrate (Millipore, WBKLS0100).

### Statistical analysis

The results of the experiments are presented as the mean ± SD. Student’s *t* test and Welch’s *t* test with unpaired two-tailed distribution were utilized to determine the statistical significance of the differences. At least three repeats of each experiment were conducted, and *p* values < 0.05 were considered significant.

## Data availability

All regarding data are available from the corresponding author.

## Supporting information

This article contains supporting information.

## Conflict of interest

The authors declare no conflict of interest with the contents of this article.
