# Supplementary material for: Fat mass and obesity-associated factor (FTO)-mediated N6-methyladenosine regulates spermatogenesis in an age-dependent manner
Source: J Biol Chem. 2023 May 3;299(6):104783. doi: 10.1016/j.jbc.2023.104783 (PMC10248873; doi:10.1016/j.jbc.2023.104783)
Supplement: Supporting Tables S1–S5 [file mmc1.docx]

**Supplementary Tables**

| **Supplementary Table S1: Characteristics of men carrying *FTO* variants.** | | |
| --- | --- | --- |
| **Characteristics** | **Patient 1** | **Patient 2** |
| Variant | p. Arg322* | p. Leu426fs |
| Age (year) | 37 | 26 |
| Semen volume (mL) | 3.7 | 5.1 |
| Sperm concentration (10^6^/mL) | 0 | 8.89 |
| Motility (%) | 0 | 10.64 |
| Progressive motility (%) | 0 | 9.57 |

| **Supplementary Table S2: Primers used for Sanger sequencing of two *FTO* variants.** | | | |
| --- | --- | --- | --- |
| **Primer Name** | **Primer Sequence (5'-3')** | **Annealing**  **Temperature (℃)** | **Product length (bp)** |
| H-FTO-p. Arg322*-F | CAAGGTAAGTATTGATTCCTTGGTT | 55 | 319 |
| H-FTO-p. Arg322*-R | TCCCATCAAATACTCTTCCAAAGC | 55 | 319 |
| H-FTO-p. Leu426fs-F | CCGGCCATTTTTGGCCATCAT | 55 | 453 |
| H-FTO-p. Leu426fs-R | ATTAATGTAGGTGCCGTGGGC | 55 | 453 |

| **Supplementary Table S3: Primers used for *Fto* KO mouse genotype analysis.** | | | |
| --- | --- | --- | --- |
| **Primer Name** | **Sequence (5'-3')** | **Annealing**  **Temperature (℃)** | **Product length (bp)** |
| M-Fto-F | CCAGTGTCTCGCATCCTCATC | 61 | 540 |
| M-Fto-R | TTACTCATCCTCAGAGCCTCAGA | 61 | 540 |

| **Supplementary Table S4: Primers used for off-target evaluation.** | | | |
| --- | --- | --- | --- |
| **Primer Name** | **Primer Sequence (5'-3')** | **Annealing**  **Temperature (℃)** | **Product length (bp)** |
| mm10-chr5:7974838-7975149-F | CGTTAGAGTTGGTGTGGGGA | 61 | 239 |
| mm10-chr5:7974838-7975149-R | GCACTGTCTACCAAGGGCTA | 61 | 239 |
| mm10-chr7:145004514-145004869-F | ACAGAATTTAGGGACCAGGGT | 61 | 299 |
| mm10-chr7:145004514-145004869-R | CAATCACCCTGTCACTTCTGT | 61 | 299 |
| mm10-chr10:4099692-4100312-F | TCATGAGTGAATCCCAGAAGG | 61 | 283 |
| mm10-chr10:4099692-4100312-R | CCTACTCATCAAATGCCACCTG | 61 | 283 |
| mm10-chr15:84202894-84203514-F | ATCAGCCCTTGTTCCTTGCA | 61 | 300 |
| mm10-chr15:84202894-84203514-R | AGCACTAGAAGCAGCAGGAT | 61 | 300 |
| mm10-chr1:174838354-174838820-F | TGGAGGACACATTCAGAGGT | 61 | 298 |
| mm10-chr1:174838354-174838820-R | TCCTCATACCCTAACACCCAC | 61 | 298 |
| mm10-chr6:37272205-37272722-F | GCAGAAATGTCAGATGGGCC | 61 | 221 |
| mm10-chr6:37272205-37272722-R | TCGGGATCTGGAATATGCTGA | 61 | 221 |
| mm10-chr16:94105980-94106669-F | GGATGCAAGCAGGACAGTTG | 61 | 221 |
| mm10-chr16:94105980-94106669-R | CTCTCCTGCCTCAAGTGTGA | 61 | 221 |
| mm10-chr10:94144485-94145174-F | GGTTCCTGTAGTCGTCAGCT | 61 | 355 |
| mm10-chr10:94144485-94145174-R | TAGCAGGCAAACTCCCATCA | 61 | 355 |
| mm10-chr3:57516317-57516834-F | AAGCCTCTTCCTCTGACTCC | 61 | 250 |
| mm10-chr3:57516317-57516834-R | GTTGATGCCGAGAGTAAAACG | 61 | 250 |
| mm10-chr2:178952627-178953144-F | GAAGCTCCCTGACTCTTGGT | 61 | 186 |
| mm10-chr2:178952627-178953144-R | ACCCTGAGCCCTTGGAGATT | 61 | 186 |

| **Supplementary Table S5: Primers used for real-time qPCR analysis.** | | | |
| --- | --- | --- | --- |
| **Primer Name** | **Primer Sequence (5'-3')** | **Annealing**  **Temperature (℃)** | **Product length (bp)** |
| M-Fto-mRNA-F | CATGAAGCGCGTCCAGAC | 60 | 252 |
| M-Fto-mRNA-R | CACCACGTCCCGAAACAAG | 60 | 252 |
