# Supplementary material for: Fat mass and obesity-associated factor (FTO)-mediated N6-methyladenosine regulates spermatogenesis in an age-dependent manner
Source: J Biol Chem. 2023 May 3;299(6):104783. doi: 10.1016/j.jbc.2023.104783 (PMC10248873; doi:10.1016/j.jbc.2023.104783)
Supplement: Supporting Figures S1–S7 [file mmc2.docx]

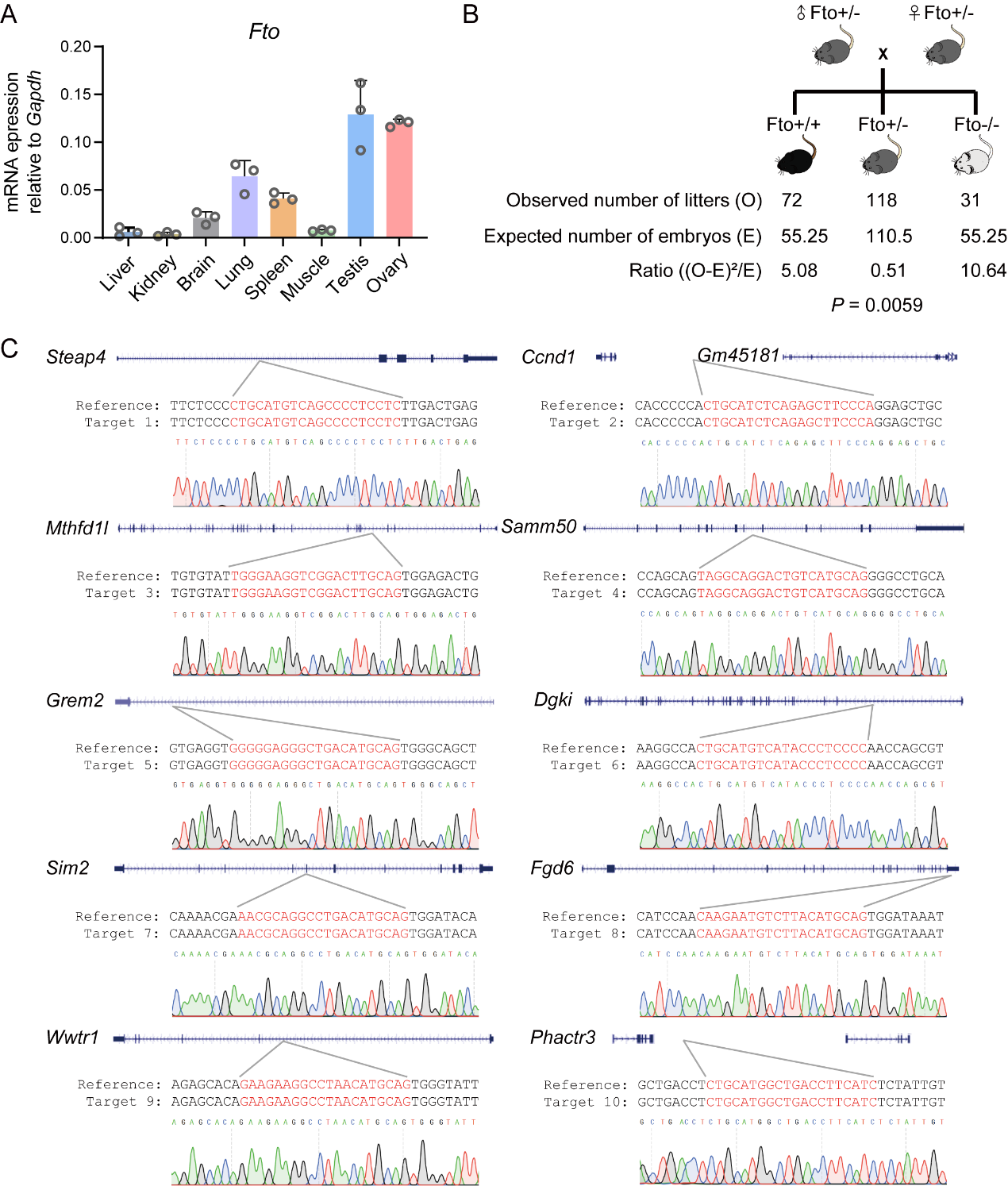


**Fig S1. Genotype frequency analysis and off-target evaluation of *Fto* KO mice**

(A) Real-time q-PCR for *Fto* transcripts in different mouse tissues with *Gapdh* as a control. Bar graphs represent the means ± SD (n = 3). (B) Genotype frequency analysis of 7-day-old offspring from *Fto* heterozygote KO mice crosses. Pearson’s Chi-squared test was used for statistical calculation. (C) The potential off-targets (predicted by Off-Spotter server) evaluation and Sanger sequencing validation of CRISPR-Cas9 in *Fto* KO mice.


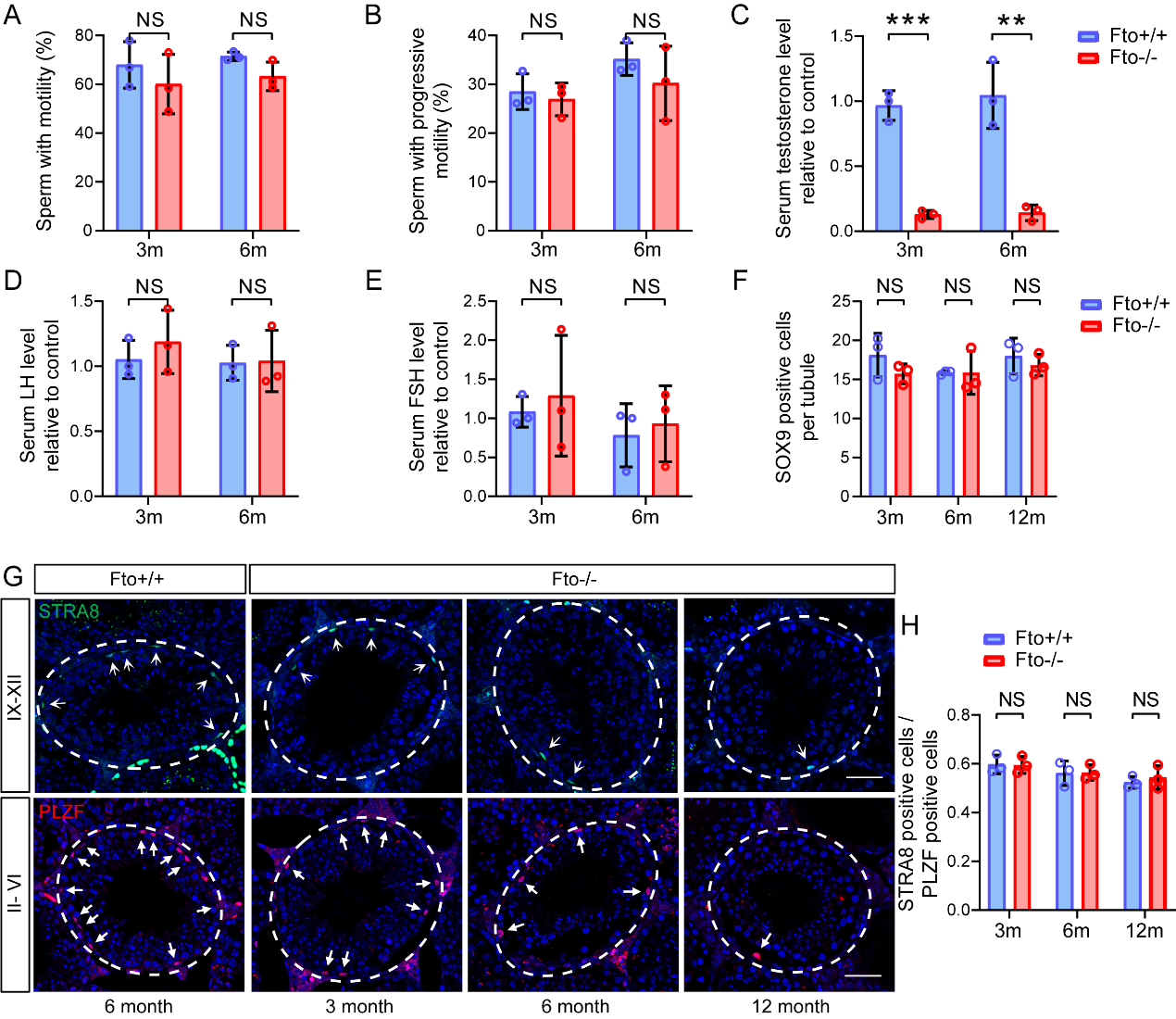


**Fig S2. Sperm mobility, hormone levels and immunofluorescence (sex determining region Y-Box 9 (SOX9) and retinoic acid 8 (STRA8)) analysis of *Fto* KO male mice**

(A-B) The percentages of sperm with motility and sperm with progressive motility from 3- and 6-month-old WT and *Fto* KO male mice were analyzed by Computer Assisted Sperm Analyzer method. Bar graphs represent the means ± SD. NS, not significant. (C-E) ELISA analysis of serum testosterone, luteinizing hormone (LH) and follicle-stimulating hormone (FSH) concentrations in 3- and 6-month-old WT and *Fto* KO male mice (n = 3). Bar graphs represent the means ± SD. NS, not significant, ***P* < 0.01, ****P* < 0.001. (F) The ratios of SOX9 positive Sertoli cells (green) per spermatogenic tubule from 3-, 6- and 12-month-old WT and *Fto* KO male mice (mice, n = 3; tubules of one mouse, n = 20). Bar graphs represent the means ± SD. NS, not significant. (G) Immunofluorescence analysis of STRA8 (green, differentiated spermatogonia marker) and promyelocytic leukemia zinc-finger (PLZF) (red, undifferentiated spermatogonia marker) in spermatogenic tubules from 3-, 6- and 12-month-old WT and *Fto* KO male mice. The light white arrows indicate STRA8 positive differentiated spermatogonia in IX-XII stages. The white arrows indicate PLZF positive undifferentiated spermatogonia in II-VI stages. Scale bars, 50 μm. (H) Comparison of the proportions between differentiated spermatogonia in IX-XII stages and undifferentiated spermatogonia in II-VI stages from the testis sections of 3-, 6- and 12-month-old WT and *Fto* KO male mice (mice, n = 3; tubules of one mouse, n = 20). Bar graphs represent the means ± SD. NS, not significant.


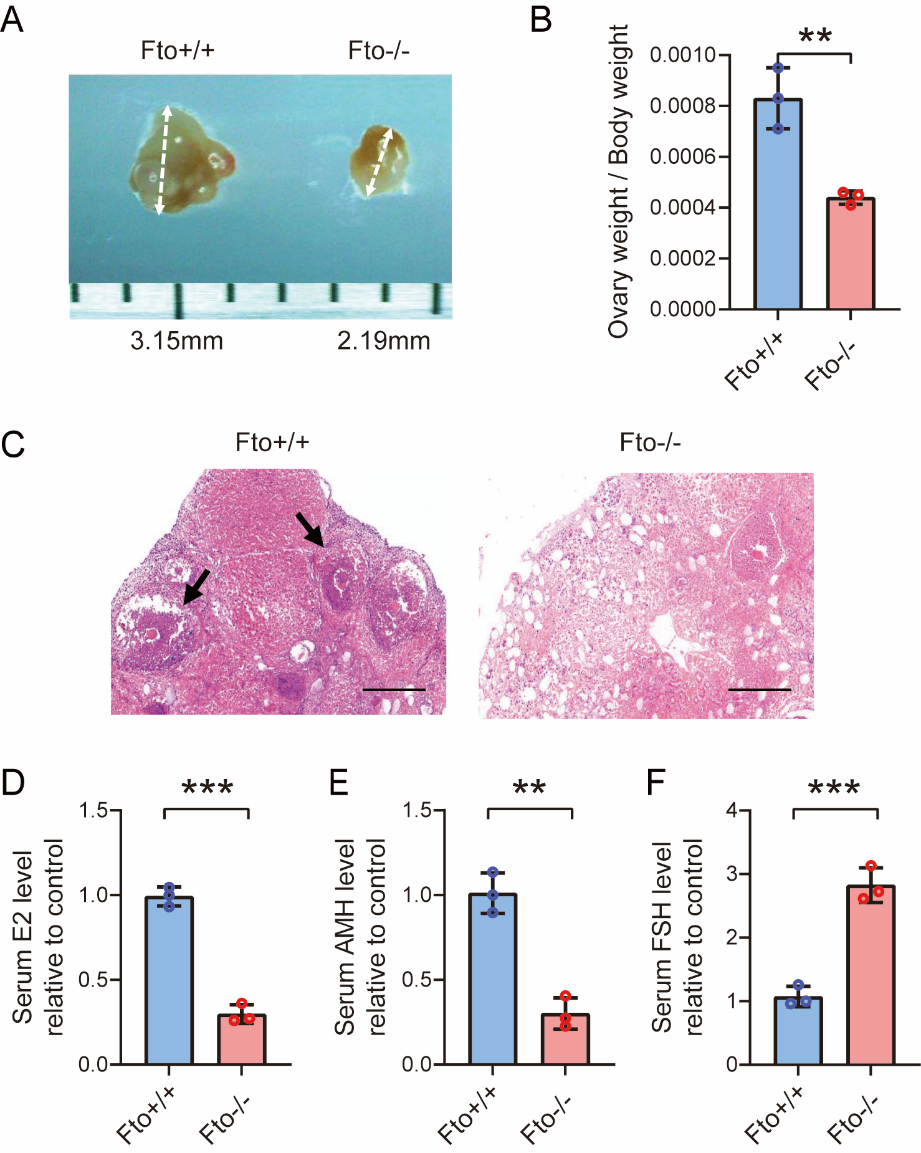


**Fig S3. Ovary histology analysis and hormone levels of *Fto* KO female mice**

(A) Morphological analysis of 6-month-old WT and *Fto* KO ovaries. The white double-sided arrows indicate the diameters of ovaries. (B) The ratios of ovary weight to body weight in 6-month-old WT and *Fto* KO ovaries (n = 3). Bar graphs represent the means ± SD. ***P* < 0.01. (C) Hematoxylin and eosin staining - stained sections of 6-month-old WT and *Fto* KO ovaries. The black arrows indicated normal follicles. Scale bars, 200 μm. (D-F) ELISA analysis of serum estradiol (E2), anti-Mullerian hormone (AMH) and FSH concentrations in 6-month-old WT and *Fto* KO female mice (n = 3). Bar graphs represent the means ± SD. ***P* < 0.01, ****P* < 0.001.


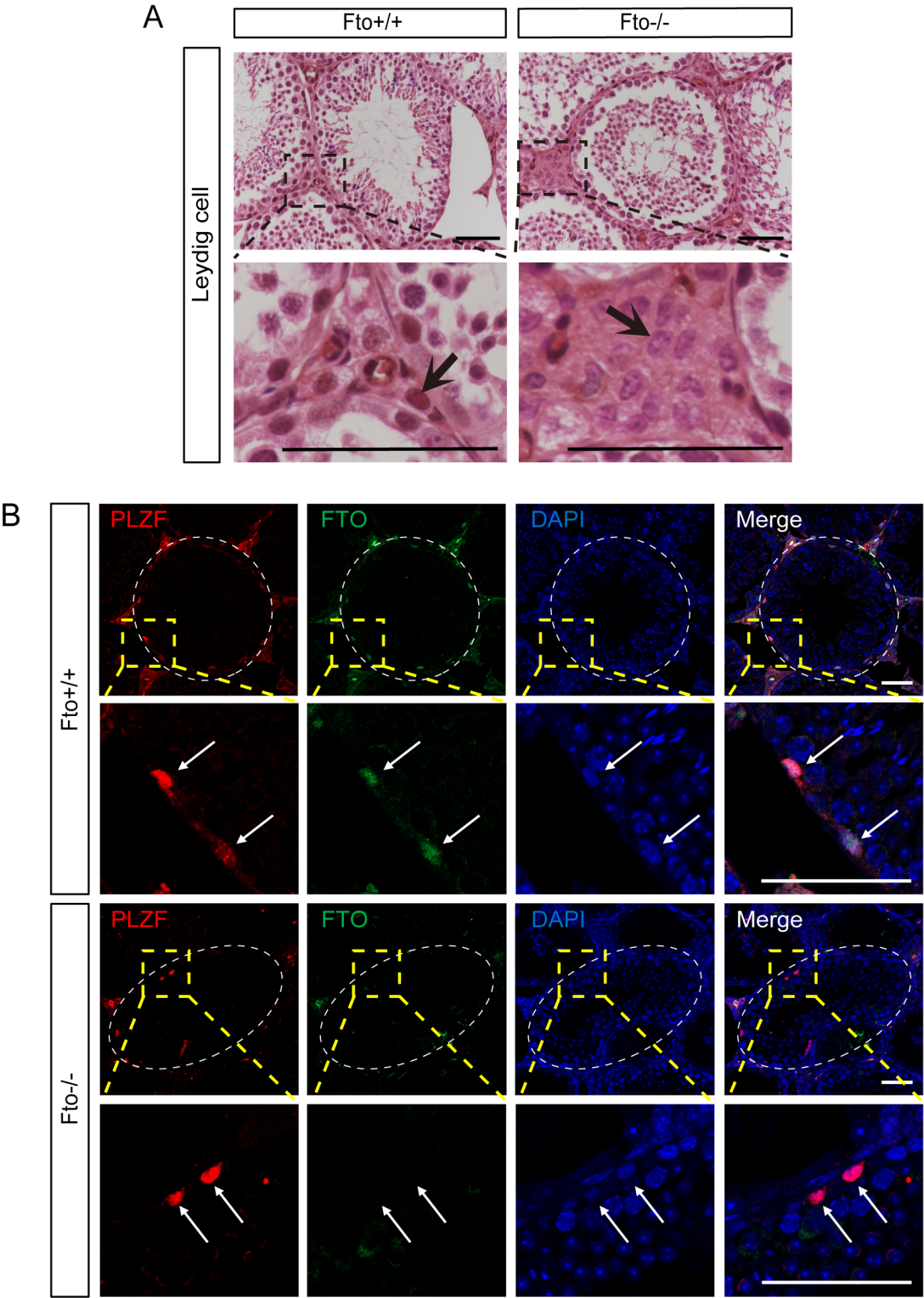


**Fig S4. FTO expression in Leydig cells and spermatogonia of WT male mice**

(A) Immunohistochemical images of FTO of testis sections from WT and *Fto* KO male mice. The black arrows indicate Leydig cells. Scale bars, 50 μm. (B) Testis sections images of FTO (green) and PLZF (red) immunostaining from WT and *Fto* KO male mice. The white arrows indicate undifferentiated spermatogonia. Scale bars, 50 μm.


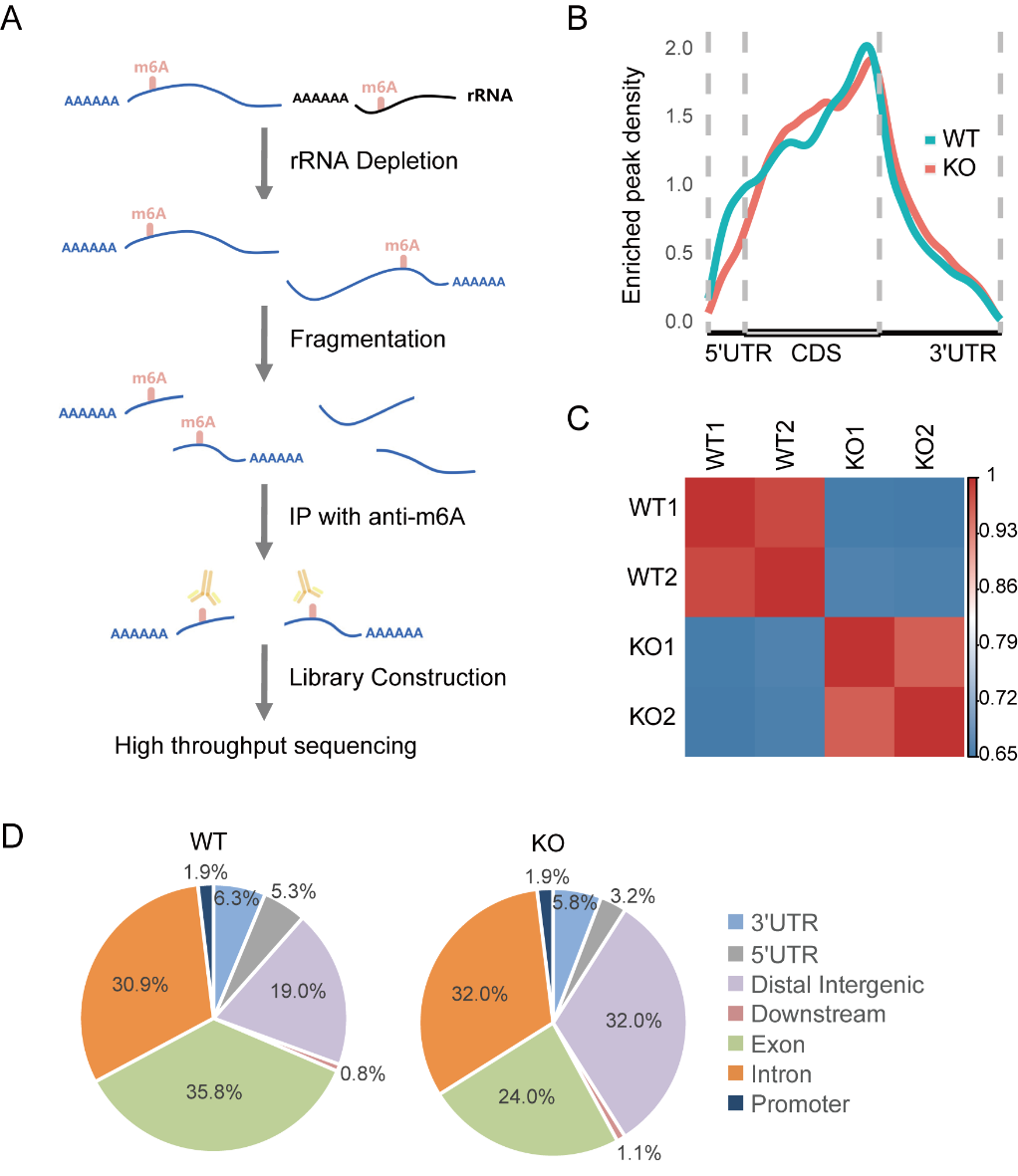


**Fig S5. The profiling of m6A modified peaks and genes in the testes of *Fto* KO mice**

(A) Schematic of m6A-MeRIP sequencing procedure. After rRNA depletion and fragmentation, RNA was analyzed through immunoprecipitation using anti-m6A antibodies. Specific bound RNA was eluted with m6A and used to construct a library for high throughput sequencing. (B) The distribution of m6A sites across the length of mRNA transcripts for 6-month-old WT and *Fto* KO mice (n = 2). (C) Heatmap of Pearson correlation on m6A level of protein-coding genes from 4 samples. (D) The fractions of m6A peaks within the 3’UTR, 5’UTR, distal intergenic regions, downstream regions, exon, intron and promoter in 6-month-old WT and *Fto* KO mice.


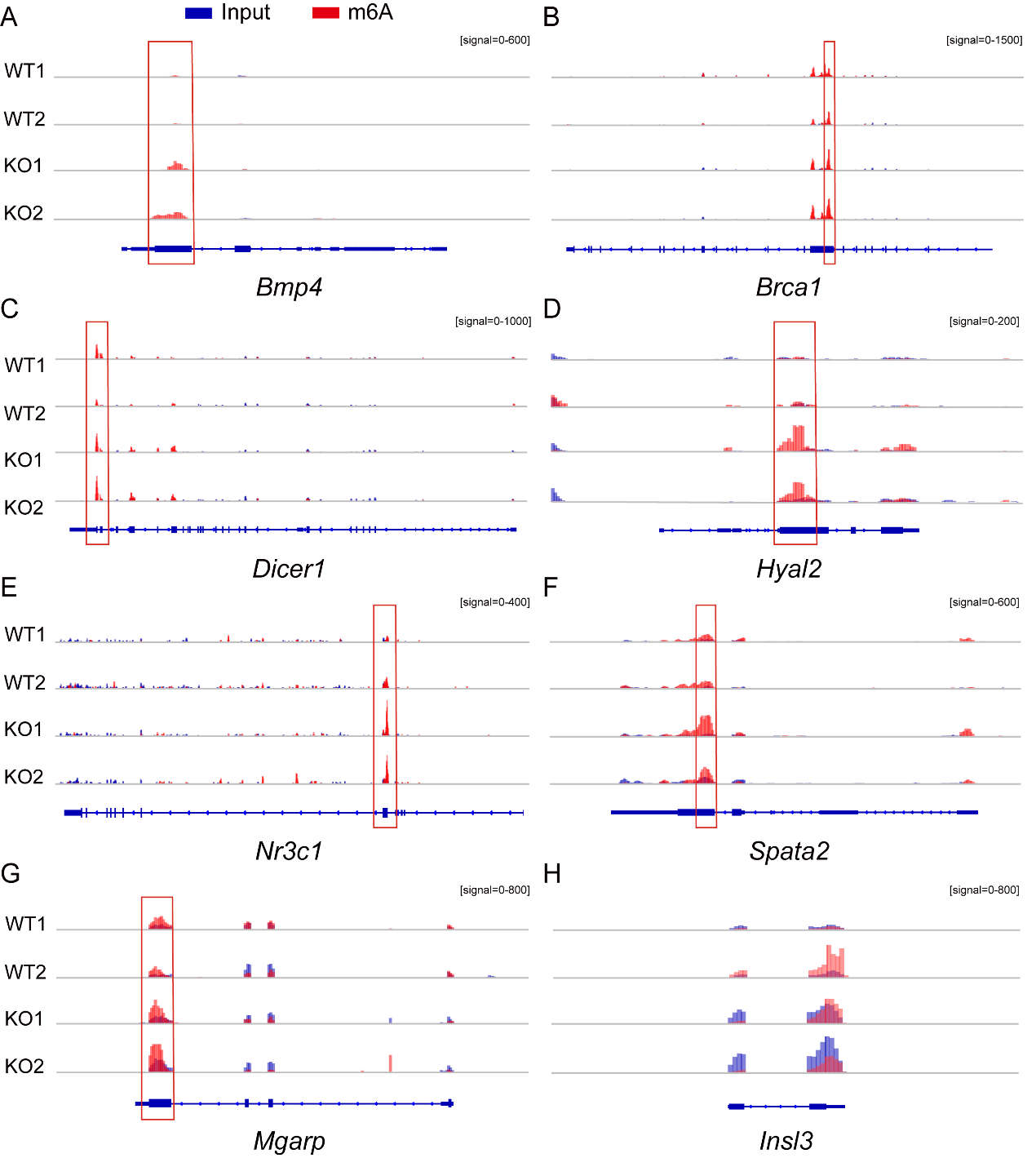


**Fig S6. The m6A abundances of FTO downstream regulatory genes**

(A-H) IGV software analysis of the m6A peaks of *Bmp4*, *Brca1*, *Dicer1*, *Hyal2*, *Nr3c1*, *Spata2,* *Mgarp* and *Insl3* mRNA transcripts in 6-month-old WT and *Fto* KO mouse testes. The red bars are m6A-modified peaks, and the blue bars are input peaks. Signal represents the abundance of input or m6A. The m6A abundances is assessed by the difference between the signals of m6A-modified peaks and input peaks.


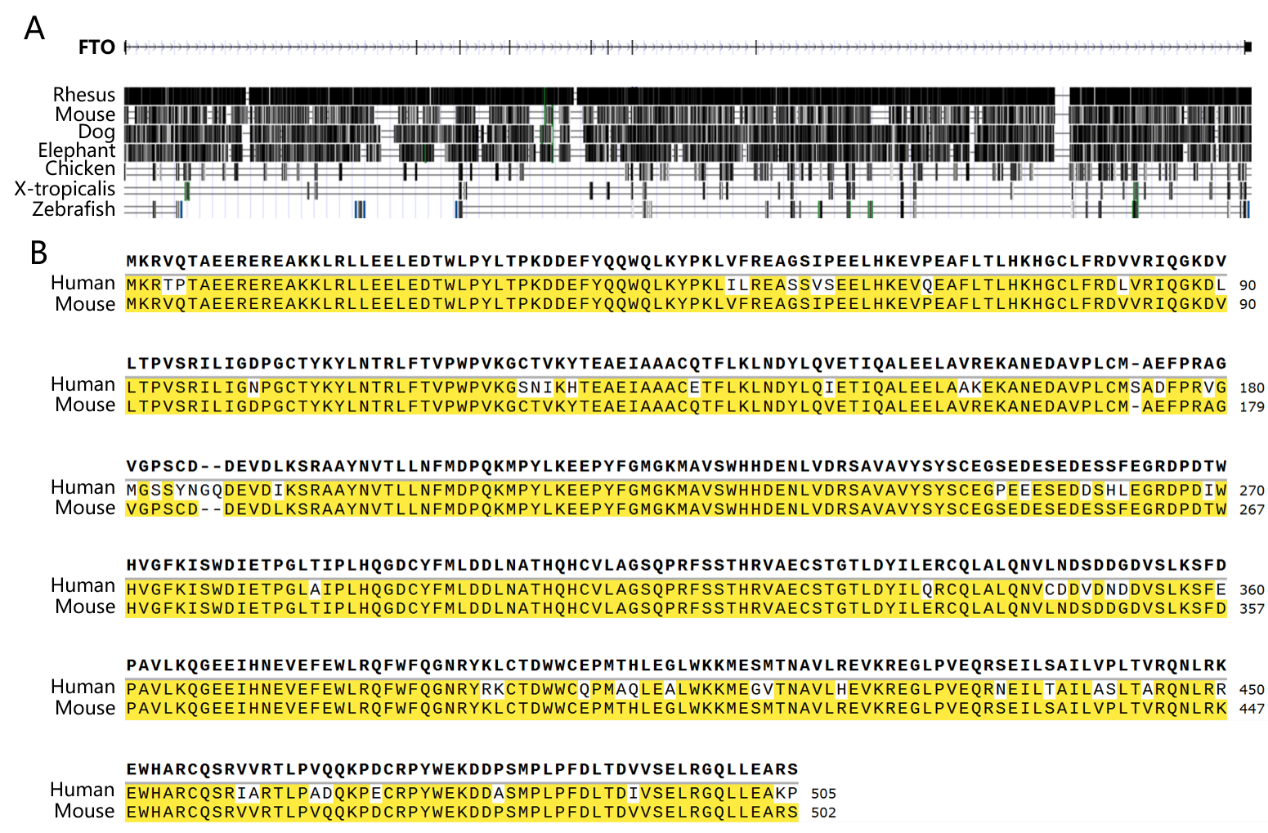


**Fig S7. Conservation analysis of FTO across different species**

(A) DNA sequence alignment shows conservation across different species according to the UCSC genome browser. (B) Amino acid sequence alignment shows conservation between human and mouse according to the UniProt server.
